# Human Growth

**Published:** 1876-05

**Authors:** 


### HUMAN GROWTH.

FROM the mechanism of a mite, to that of a man, there are inherent evidences of the same great Creating Mind—great in Wisdom, great in Power, and great in His Beneficence. Trees grow most in summer-time, and so do men. In summer, there is warmth, relaxation, opening, budding out—there is growth; in winter there is the struggle for life—the great manufactories of the system have to do increased work, in order to keep the body warm. It is often so cold in winter, that most of a farmer's time during the day, is expended in keeping up the fires

It is the same in the human body: extra labor must be done by the multitudinous workmen, whose business it is to keep the wheels of life in motion. In winter, we eat a fourth more, and require more sleep, by a full hour, in the twenty-four. So that he who is so systematic as to go to bed at the same hour, and leave it the same hour, the year round, does a violence to his constitution, which will tell undeniably in the direction of debility and premature decay.

The "stripling" and the "sapling" "spread out" luxuriantly; but as the time of the "sear and yellow leaf" comes on, their growth becomes more and more feeble, then ceases, and they die! The hair grows fastest in the summer, and in the young. A finger nail is renewed in a hundred and thirty-two days in winter, but requires only a hundred and sixteen of warm weather. And as light hastens vegetation, so it is known that the hair grows faster in the day time than in the night; and the beautiful principle holds good as to our moral being. We all expand and grow into the likeness of our Great Father, in proportion as charity keeps up the warm summer time in our hearts—while the sun-light of a life that is pure and true, dispels the clouds and darkness of wrong-doing, and creates an atmosphere fit for the breath of angels.

